# Trauma-associated extracellular histones mediate inflammation *via* a MYD88-IRAK1-ERK signaling axis and induce lytic cell death in human adipocytes

**DOI:** 10.1038/s41419-024-06676-9

**Published:** 2024-04-23

**Authors:** Julian Roos, Julia Zinngrebe, Markus Huber-Lang, Ludmila Lupu, Miriam A. Schmidt, Hannah Strobel, Mike-Andrew Westhoff, Ulrich Stifel, Florian Gebhard, Martin Wabitsch, Tom Eirik Mollnes, Klaus-Michael Debatin, Rebecca Halbgebauer, Pamela Fischer-Posovszky

**Affiliations:** 1 Department of Pediatrics and Adolescent Medicine, University Medical Center, Ulm, Germany; 2 Institute of Clinical and Experimental Trauma Immunology, University Medical Center, Ulm, Germany; 3 Department of Orthopedic Trauma, Hand, and Reconstructive Surgery, University Medical Center, Ulm, Germany; 4 Division of Pediatric Endocrinology and Diabetes, Department of Pediatrics and Adolescent Medicine, University Medical Center, Ulm, Germany; 5 Department of Immunology, Oslo University Hospital and University of Oslo, Oslo, Norway; 6 Research Laboratory, Nordland Hospital Trust, Bodo, Norway

**Keywords:** Preclinical research, Acute inflammation, Apoptosis

## Abstract

Despite advances in the treatment and care of severe physical injuries, trauma remains one of the main reasons for disability-adjusted life years worldwide. Trauma patients often suffer from disturbances in energy utilization and metabolic dysfunction, including hyperglycemia and increased insulin resistance. White adipose tissue plays an essential role in the regulation of energy homeostasis and is frequently implicated in traumatic injury due to its ubiquitous body distribution but remains poorly studied. Initial triggers of the trauma response are mainly damage-associated molecular patterns (DAMPs) such as histones. We hypothesized that DAMP-induced adipose tissue inflammation contributes to metabolic dysfunction in trauma patients. Therefore, we investigated whether histone release during traumatic injury affects adipose tissue. Making use of a murine polytrauma model with hemorrhagic shock, we found increased serum levels of histones accompanied by an inflammatory response in white adipose tissue. In vitro, extracellular histones induced an inflammatory response in human adipocytes. On the molecular level, this inflammatory response was mediated *via* a MYD88-IRAK1-ERK signaling axis as demonstrated by pharmacological and genetic inhibition. Histones also induced lytic cell death executed independently of caspases and RIPK1 activity. Importantly, we detected increased histone levels in the bloodstream of patients after polytrauma. Such patients might benefit from a therapy consisting of activated protein C and the FDA-approved ERK inhibitor trametinib, as this combination effectively prevented histone-mediated effects on both, inflammatory gene activation and cell death in adipocytes. Preventing adipose tissue inflammation and adipocyte death in patients with polytrauma could help minimize posttraumatic metabolic dysfunction.

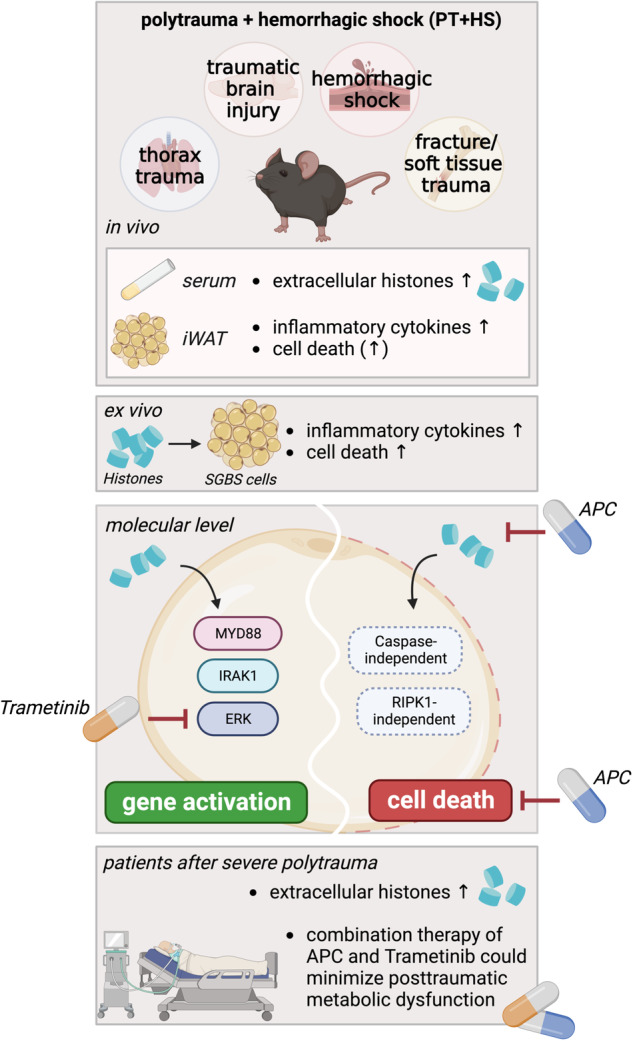

## Introduction

Globally, millions of people die from physical injuries every year [1, 2]. Although some decrease in injuries has been observed in the last decades worldwide, trauma remains an important cause of morbidity and mortality in both, developed and developing countries [1]. In 2017, one tenth of the global burden of disease was caused by injuries [2]. Severe tissue trauma can induce a systemic inflammatory response syndrome (SIRS) potentially culminating in organ dysfunction and even failure [3, 4]. The more severe the SIRS, the higher the mortality of trauma patients [5]. In recent years, great efforts have been made to improve the prognosis and outcome of patients with trauma. Nevertheless, trauma consequences remain a medical and societal challenge.

Tissue damage during trauma results in the release of a variety of damage-associated molecular patterns (DAMPs) [3, 6]. These DAMPs include nucleosomes and histones which have been attributed an important role in mediating traumatic SIRS [7, 8]. Histones are basic proteins that bind DNA in the nucleus and play an essential role in chromatin condensation. Outside the cell, however, they can cause considerable harm as injection of histones into mice [9] or release of histones during sepsis [10] resulted in endothelial damage and multiple organ failure. Moreover, increased appearance of extracellular histones is associated with injury severity, acute lung injury, multiorgan failure and mortality in critically injured patients [11]. Circulating free histones and nucleosomes *i.e*., histones in complex with DNA, were increased and associated with acute lung injury in patients with severe non-thoracic blunt trauma [12]. This fact illustrates that circulating histones even induce damage of remote organs not directly affected by the traumatic impact vector itself.

Trauma patients can suffer from severe metabolic changes such as hyperglycemia and increased insulin resistance [13, 14]. White adipose tissue (WAT) represents the largest organ of the human body and is an essential regulator of energy homeostasis. Furthermore, it functions as an important endocrine organ with crosstalk to *e.g*., liver, muscle, bone, and brain, thereby controlling vital processes such as food intake, insulin sensitivity, and blood pressure [15, 16]. WAT is found in different parts of the human body: while subcutaneous WAT forms an insulating layer under the skin, visceral WAT coats and secures internal organs. WAT has important mechanical properties and protects parts of the human body exposed to high levels of mechanical stress [16]. Under physiological conditions, WAT stores excess energy as triglycerides. However, little is known about the role of adipose tissue during trauma although almost any traumatic injury causes (co-)damage of WAT. Furthermore, as WAT is strongly supplied with blood, it might even be affected by DAMP release at distant sites.

In this study we utilized a murine model of polytrauma with hemorrhagic shock (PT + HS) and demonstrate increased histone serum levels as well as upregulated inflammatory gene expression in WAT. By performing a detailed in vitro analysis of the response of WAT to extracellular histones in model systems of white adipocytes we provide characterization of the combined impact of histone-induced gene activation and cell death in WAT during trauma on the molecular and functional level. The results presented here may have implications for trauma and critically ill patients suffering from trauma-induced metabolic dysfunction with hyperglycemia and increased insulin resistance.

## Materials and methods

### Antibodies and reagents

Histones (H9250), cycloheximide (239763) and 7-Cl-O-Nec-1 (504297) were purchased from Sigma-Aldrich (Taufkirchen, Germany), zVAD.fmk from Bachem (Bubendorf, Switzerland), trametinib (GSK1120212) and dinaciclib (S2768) from Selleckchem (Planegg, Germany), TNF (300-01A) from Peprotech (Cranbury, NJ) and Human Activated Protein C (APC) (RP-43095) from Thermo Fisher Scientific (Waltham, MA). The following antibodies were used: cleaved Caspase 3 (#9661, Cell Signaling, Frankfurt am Main, Germany), Caspase-8 (#ALX-804-242-C100, Enzo Life Sciences, Farmingdale, NY), PARP (#9542, Cell Signaling), pIκBα (#9246, Cell Signaling), IκBα (#9242, Cell Signaling), pERK (#9106, Cell Signaling), ERK (#M5670, Sigma-Aldrich), hFAB rhodamine GAPDH (#12004168, BioRad, Hercules, CA), StarBright Blue 520 Goat Anti-Mouse IgG (#12005867), StarBright Blue 700 Goat Anti-Mouse IgG (#12004158), StarBright Blue 520 Goat Anti-Rabbit IgG (#12005870), StarBright Blue 700 Goat Anti-Rabbit IgG (#12004162) (all from BioRad), HRP-labelled goat anti-rabbit Ig (#4010-05, Southern Biotech, Birmingham, AL), HRP-labelled goat anti-mouse IgG2b (#1090-05, Southern Biotech).

### Animal experiments

Male C57BL/6 and B6.129S4-Cd14^tm1Frm^/J mice [17] were obtained from Jackson Laboratories, Bar Harbor, ME, and bred at the animal facility of Oslo University. All animals were genotyped before being transferred to Ulm University Medical Center at least 10 days before starting the experiment. Power analysis was performed before conduction of the study to calculate the appropriate group size. Animals were used at an age of 10-12 weeks. The study protocol was approved by the Federal Authorities for animal research, Tübingen, Germany. All experiments followed the NIH Guidelines for the use of laboratory animals. Animals had free access to food and water.

Mice were randomly assigned to sham treatment (*n* = 7-8) or polytrauma and hemorrhagic shock (*n* = 8) as described previously [18, 19]. Anesthesia was initialized with 2.5% sevoflurane (Abbott, Wiesbaden, Germany) in oxygen and sustained during the entire experiment. Briefly, polytrauma consisted of thoracic trauma, closed head injury, and femur fracture including soft tissue injury [20]. Pressure controlled hemorrhage was induced via a microcatheter in the femoral artery and a mean arterial pressure of 30 mmHg (±5 mmHg) was maintained for 60 min. Afterwards, animals were resuscitated with a balanced electrolyte solution and monitored until inguinal and epididymal adipose tissue pads were removed 4 h after trauma. All animals were included in further analyses once they had completed the trauma/sham protocol with the exception of one animal in the treatment group where the availability of inguinal WAT was limited and, which, thus, could not be analyzed by TUNEL assay. Due to the nature of the animal experiment, investigators were not blinded during the animal study protocol. However, investigators were blinded to the group allocation during subsequent experimental procedures.

### Histological analysis

Adipose tissue was fixed in 4% formalin, paraffin-embedded, cut into 4 µm sections and stained with hematoxylin and eosin (H&E). Staining of apoptotic cells was performed using the In Situ Cell Death Detection Kit (Roche Diagnostics GmbH, Mannheim, Germany) and nuclei were counterstained with DAPI. Images were taken using an Axio Imager A1 microscope (Zeiss, Oberkochen, Germany). For evaluation of adipocyte apoptosis, the percentage of TUNEL-positive adipocyte nuclei was assessed by a blinded observer in a minimum of six fields of view per animal in 100-fold magnification.

### Clinical study

A post-hoc analysis of plasma samples from a monocentered observational study in multiply injured patients and healthy volunteers [21] was performed. Patients were included upon arrival at the emergency department of Ulm University Medical Center if their Injury Severity Score was 25 or higher, and blood was drawn repeatedly during the posttraumatic course.

### Nucleosome quantification

Free nucleosomes in human and murine plasma samples were measured using the Cell Death Detection ELISA^PLUS^ (Roche) according to the manufacturer’s instructions and normalized to total plasma protein concentrations. According to the manufacturer the kit quantifies relative amounts of histone-complexed DNA fragments (mono- and oligonucleosomes): “Anti-histone reacts with the histones H1, H2A, H2B, H3 and H4 of various species and anti-DNA binds to single and double-stranded DNA. The ELISA therefore enables the detection of mono- and oligonucleosomes of different species” (https://www.sigmaaldrich.com/DE/en/product/roche/celldethro).

### Cell culture

Simpson-Golabi-Behmel syndrome (SGBS) preadipocytes [22–24] and human multipotent adipose-derived stem cells (hMADS) [25] were cultured as previously described [24]. Cell lines have not recently been authenticated by STR-profiling or tested for mycoplasma contamination. Differentiation was induced using an adipogenic induction cocktail containing FBS-free DMEM-F12 (Thermo Fisher Scientific) supplemented with 10 μg/ml transferrin, 20 nM insulin, 100 nM cortisol, 200 pM T3, 25 nM dexamethasone, 250 μM IBMX and 2 μM rosiglitazone (all from Sigma-Aldrich) as described [24]. Four days later, medium was changed into FBS-free DMEM-F12 supplemented with 10 μg/ml transferrin, 20 nM insulin, 100 nM cortisol, 200 pM T3. Nalm6 cells were cultured in RPMI supplemented with 20% FBS and 1% L-Glutamine and split twice per week at a ratio of 1:20.

### siRNA transfection

For gene knockdown studies, SGBS cells were transfected with 20 nM Dharmacon siGENOME siRNA targeting IRAK1 (M-004760-03-0005) or MYD88 (M-004769-01-0005) or non-targeting control (siGENOME Non-Targeting Control siRNA Pool #2) (all Horizon, Lafayette, CO) mixed with 0.66 μl/cm^2^ Lipofectamine 2000 or Lipofectamine RNAiMAX (Thermo Fisher Scientific) according to the manufacturer’s protocol.

### RNA isolation, reverse transcription and quantitative PCR (qPCR)

Total RNA was isolated with Direct-zol RNA mini Prep Kit (Zymo Research, Freiburg im Breisgau, Germany) according to the manufacturer’s protocol after cells were harvested with Tri-Reagent (Zymo Research). Whole tissues were minced in liquid nitrogen and afterwards resolved in Tri-Reagent. 100 ng to 1 µg RNA were reverse transcribed with SuperScript II Reverse Transcriptase (Thermo Fisher Scientific).

For mRNA quantification the SsoAdvanced Universal SYBR Green Supermix was used on a CFX Connect plate cycler (BioRad) using the primers given below. Results were normalized to the housekeeping gene Hypoxanthin-Guanin-Phosphoribosyltransferase (HPRT) using the 2^−ΔCt^ method [26].

The following murine primer sequences were used (5′ > 3′): Hprt-fwd: GCTGGTGAAAAGGACCTC, Hprt-rev: CACAGGACTAGAACACCT; Il6-fwd: GAT GGA TGC TAC CAA ACT GGA, Il6-rev: TCT GAA GGA CTC TGG CTT TG; Cxcl1-fwd: CTG GGA TTC ACC TCA AGA ACA TC, Cxcl1-rev: CAG GGT CAA GGC AAG CCT C; Tnf-fwd: CCA GAC CCT CAC ACT CAG ATC ATC TTC TC, Tnf-rev: CTA GTT GGT TGT CTT TGA GAT CCA TGC CGT; Mcp1-fwd: AGG TCC CTG TCA TGC TTC TG, Mcp1-rev: GGG ATC ATC TTG CTG GTG AA; Adipoq-fwd: GTT CCT CTT AAT CCT GCC CAG TCA TGC C, Adipoq-rev: GGA CCA AGA AGA CCT GCA TCT CCT TTC TC; Leptin-fwd: GAG ACC CCT GTG TCG GTT C, Leptin-rev: CTG CGT GTG TGA AAT GTC ATT G.

The following human primer sequences were used (5′ > 3′): ADIPOQ-FWD: GGC CGT GAT GGC AGA GAT, ADIPOQ-REV: CTT CAG CCC CGG GTA CT; HPRT-FWD: GAG ATG GGA GGC CAT CAC ATT GTA GCC CTC, HPRT-REV: CTC CAC CAA TTA CTT TTA TGT CCC CTG TTG ACT GGT C; IL6-FWD: TAC CCC CAG GAG AAG ATT CC, IL6-REV: TTT TCT GCC AGT GCC TCT TT; IL8-FWD: TGC CAA GGA GTG CTA AAG AAC TTA GAT GTC AG, IL8-REV: AGC TTT ACA ATA ATT TCT GTG TTG GCG CAG TG; MCP1-FWD: TCC CAA AGA AGC TGT GAT CTT CAA GAC C, MCP1-REV: AGT GAG TGT TCA AGT CTT CGG AGT TTG G; MYD88-FWD: GGC TGC TCT CAA CAT GCG A, MYD88-REV: CTG TGT CCG CA CGT TCA AGA; all from Biomers, Ulm, Germany.

### Enzyme-linked immunosorbent assay (ELISA)

Adipokines in SGBS supernatant after histone stimulation were determined using the Human IL-8/CXCL8 DuoSet ELISA and the Human Adiponectin/Acrp30 DuoSet ELISA (both R&D Systems, Abingdon, UK) and the BD OptEIA™ Human IL-6 ELISA Set (BD Biosciences, San Diego, CA) according to manufacturers’ instructions.

### Western blot

Protein was harvested and SDS-PAGE and protein transfer were performed as described previously [27]. Images were captured with the ChemidocTM MP (BioRad) imaging system. Uncropped images can be found in the Appendix (Supplementary Information).

### TUNEL staining

SGBS cells were seeded on chamber slides (BD Bioscience) and differentiated into adipocytes. On days 10–14, cells were stimulated with 25 or 50 µg/ml histones or left untreated (control) for 16 h prior to staining with the Click-iT™ Plus TUNEL-Assay kit (Thermo Fisher) according to the manufacturer’s instructions. Slides were co-stained with DAPI (Sigma-Aldrich) prior to imaging on the BZ-9000 microscope (Keyence, Osaka, Japan). TUNEL-positive cells were determined using ImageJ Fiji (version 1.54f). TUNEL fluorescence intensity was quantified per nucleus and the percentage of positive nuclei normalized to the respective control.

### Propidium iodide staining

25 µg/ml propidium iodide solution (Sigma-Aldrich), 1:1000 diluted BODIPY 493/503 (Thermo Fisher) and 0.5 µM Hoechst33342 (Thermo Fisher) were added to the wells for 10 min after 4 or 24 h of histone stimulation. Microphotographs were taken using the BZ-9000 (Keyence) microscope.

### Cell viability assay

The CellTiter-Glo® Luminescent Cell Viability Assay (Promega, Walldorf, Germany) was used according to the manufacturer’s instructions.

### Generation of macrophage-conditioned medium

SGBS adipocytes were stimulated with macrophage-conditioned medium as positive control following a previously established protocol [28]. In brief, human THP-1 monocytes were stimulated with 125 ng/ml PMA to differentiate them into macrophages. Conditioned media were collected from 1 million cells/ml incubated in RPMI medium supplemented with 0.1% BSA for 48 h. Adipocytes were treated with 10% macrophage-conditioned medium (Fig. 3F, G).

### DNA fragmentation assay

Quantitative determination of DNA fragmentation was performed with SGBS adipocytes [42]. In brief, 4 h after histone stimulation, 100 µl of sixfold concentrated hypotonic fluorochrome solution (250 μg/ml propidium iodide, 1.2% sodium citrate, and 1.2% Triton-X 100) were added to 500 µl stimulation media. After lysis at 4° C for 1 h, cells were resuspended and analyzed by flow cytometry.

### Annexin-V/PI staining

SGBS cells on days four to six of adipogenic differentiation were stimulated with 50 µg/ml histones and assessed for Annexin-V/propidium iodide (PI) positivity by flow cytometry. In brief, cells were harvested by trypsinization, centrifuged, washed once with PBS, centrifuged and resuspended in Annexin-V buffer (0.01 M HEPES (Sigma-Aldrich) in Sterofundin) containing Annexin-V-FITC from Roche at a dilution of 1:100. Cells were stained for 25 min at room temperature protected from light. Directly before sample acquisition at the Attune NxT Flow Cytometer PI was added at a final concentration of 1 µg/ml.

### Lactate dehydrogenase (LDH) release assay

To quantify LDH release in media supernatant the LDH-Glo™ Cytotoxicity Assay (Promega) was used according to the manufacturer’s protocol.

### Statistics

No statistical methods were used to pre-determine sample size for in vitro experiments. In vitro experiments were performed at least three times. GraphPad Prism (version 9.4.1, GraphPad Software, La Jolla, CA) software was used to perform statistical analyses. The statistical tests applied are provided for each figure in the corresponding figure legend.

## Results

### Polytrauma with hemorrhagic shock induces an inflammatory response in murine WAT

**Fig. 1:**
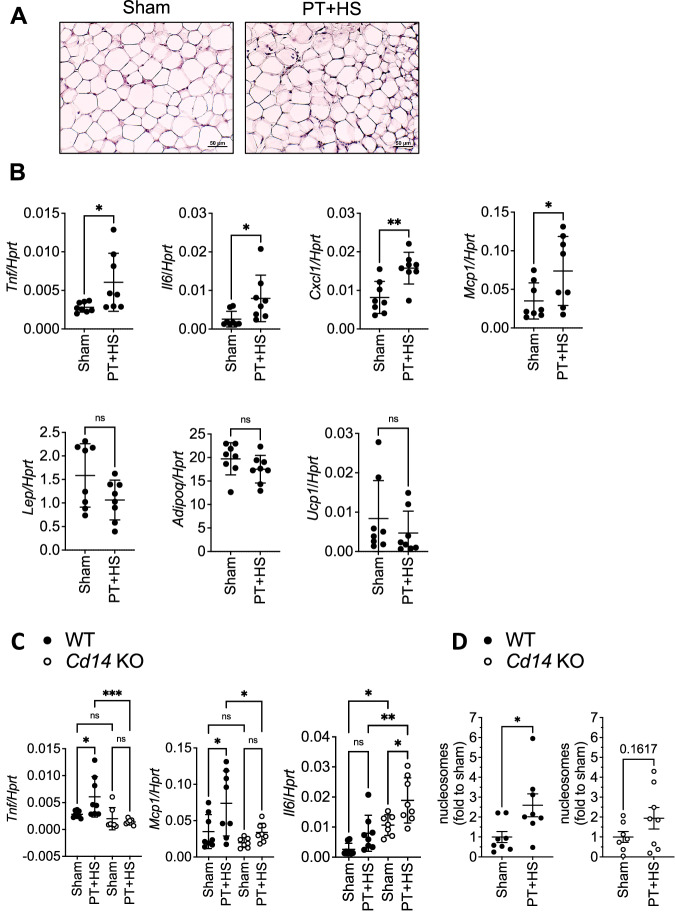
Polytrauma with hemorrhagic shock induces an inflammatory response in murine WAT. **A**, **B** Inguinal WAT (iWAT) was collected from mice 4 h after polytrauma and hemorrhagic shock (PT + HS) or from sham-treated (Sham) mice. Representative H&E-stained tissue sections from inguinal WAT are depicted (**A**). *Tnf*, *Il6*, *Cxcl1*, *Mcp1*, *Lep*, *Adipoq* and *Ucp1* mRNA expression from iWAT is shown in relation to *Hprt* (2^−ΔCt^) (**B**). **C** Expression of *Tnf*, *Il6*, and *Mcp1* mRNA was determined in iWAT of *Cd14*-deficient mice after PT + HS or from sham-treated mice, and compared to iWAT of WT mice (from **B**). **D** Free serum nucleosomes (histones) were determined in mice with and without PT + HS. Data are displayed as single values and mean ± SEM of *n* = 8 WT mice per group (**B**–**D**) or *n* = 7–8 *Cd14* KO mice (**C**, **D**) per group. Unpaired two-sided *t*-test (**B**, **D**), ordinary two-way ANOVA with main effects only, Šídák’s multiple comparison’s test (**C**), *p < 0.05, **p < 0.01, ***p < 0.001, *ns* non-significant.

To investigate whether inflammatory changes are detectable in WAT after trauma, we took advantage of a murine polytrauma (PT) model consisting of traumatic brain injury (TBI), thoracic trauma, and fracture/soft tissue injury combined with hemorrhagic shock (HS) [18]. Four hours after trauma induction, the inguinal WAT (iWAT) and gonadal WAT (gWAT) depots were dissected from sites not directly hit by the traumatic force vectors. Here, the overall WAT morphology was not altered in traumatized as compared to sham mice (Fig. 1A, Supplementary Fig. 1A). Next, we assessed whether PT + HS resulted in increased remote adipose tissue inflammation. We found mRNA expression of the inflammatory cytokines TNF (gene: *Tnf*) and IL-6 (gene: *Il6*), and of the chemokines (C-X-C motif) ligand (CXCL) 1 (gene: *Cxcl1*) and MCP-1 (gene: *Mcp1*) to be significantly increased in iWAT of the trauma group, whereas the classical adipokines adiponectin (gene: *Adipoq*) and leptin (gene: *Lep*) were not altered (Fig. 1B). The browning marker uncoupling protein 1 (UCP1; gene: *Ucp1*) was found to be increased in WAT after surgical trauma [29] yet not altered in iWAT (Fig. 1B). In gWAT, both, inflammatory marker gene expression as well as *Adipoq* and *Lep* mRNA expression were not significantly different (Supplementary Fig. 1B).

Although the iWAT depots were not directly hit by the traumatic vector itself they nevertheless showed an inflammatory response upon trauma. Thus, we reasoned that DAMPs might be released from the traumatic sites *via* the bloodstream thereby affecting iWAT depots. As DAMPs can be sensed by toll-like receptors (TLRs), we next assessed whether TLRs might play a role in mediating the inflammatory response in iWAT depots upon trauma. To interfere with the TLR signaling cascade, we studied the effects of PT + HS in mice carrying a knockout of cluster of differentiation (CD) 14, which is a co-receptor of several TLRs, including TLR2 and TLR4, promoting their intracellular signaling [30–34]. CD14 is expressed by monocytes/macrophages and, to a lesser extent, also by fibroblasts and adipocytes (see The Human Protein Atlas (proteinatlas.org)). The upregulation of *Tnf* and *Mcp1* by PT + HS was completely blunted in iWAT of *Cd14*^−/−^ mice compared to wild-type (WT) mice (Fig. 1C; data of the *Cd14*^−/−^ mice was compared to data generated in WT mice shown in detail also in Fig. 1B). Of note, *Il6* was significantly higher expressed in iWAT of *Cd14*^*−/−*^ mice compared to controls under sham conditions, and the levels further increased with PT + HS (Fig. 1C) suggesting that CD14 is a negative regulator of *Il6* expression in iWAT. *Lep* and *Cxcl1* mRNA expression were not affected by absence of CD14 (Supplementary Fig. 1C). In summary, this set of data demonstrates that PT + HS induce a pro-inflammatory response in iWAT mediated, at least in part, by the TLR co-receptor CD14. This suggests TLRs to play a role in trauma-induced adipose tissue inflammation.

DAMPs such as extracellular histones were recently shown to exhibit an important role in trauma-mediated inflammatory signaling and cell death induction [9, 35, 36]. Thus, we measured circulating histones in the form of nucleosomes in our mouse model (Fig. 1D). In line with our hypothesis, circulating histones were increased in WT or *Cd14*^−/−^ mice with PT + HS as compared to sham-treated mice (Fig. 1D) suggesting that release of histones during trauma could potentially affect distant organs, such as WAT, *via* the circulation.

### Extracellular histones induce an inflammatory response in human white adipocytes

**Fig. 2:**
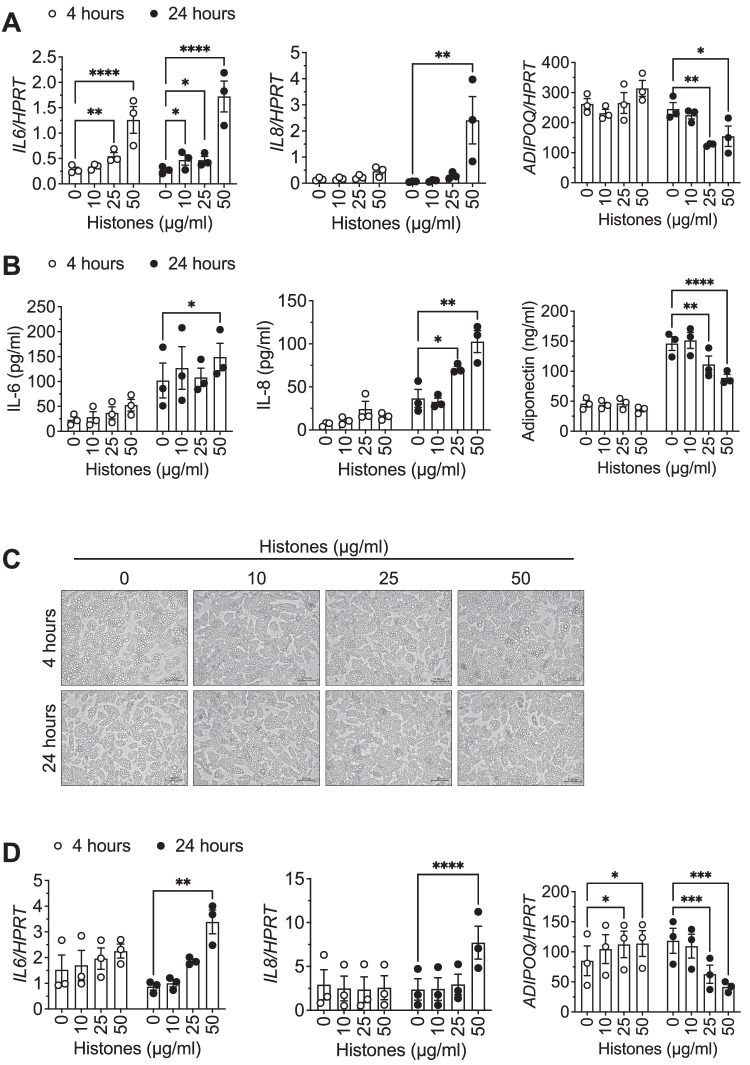
Extracellular histones induce inflammation in human white adipocytes. SGBS (**A**–**C**) or hMADS (**D**) adipocytes were stimulated for 4 or 24 h with increasing concentrations of histones. **A** *IL6*, *IL8* and *ADIPOQ* mRNA expression in relation to *HPRT* (2^−ΔCt^) is shown. **B** Concentration of IL-6, IL-8 and adiponectin in media supernatant after 4 and 24 h of histone stimulation was determined. **C** Representative microphotographs (scale bar: 100 µM) of SGBS cells upon histone stimulation are depicted. **D** *IL6*, *IL8* and *ADIPOQ* mRNA expression in relation to *HPRT* (2^−ΔCt^) is shown. Data are displayed as mean ± SEM of three independent experiments performed in duplicates (**A**, **D**) or triplicates (**B**). Repeated measures two-way ANOVA with Dunnett correction for multiple comparisons. *p < 0.05, **p < 0.01, ***p < 0.001, ****p < 0.0001.

We next investigated whether trauma-induced histone release could directly affect white adipocytes and used human Simpson-Golabi-Behmel syndrome (SGBS) cells as a model system [22–24]. These cells show a high adipogenic differentiation capacity and display the typical functional adipocyte features such as insulin-stimulated glucose uptake, beta-adrenergic-stimulated lipolysis, or secretion of adipokines [22–24]. SGBS adipocytes were stimulated with increasing histone concentrations ranging from 10–50 µg/ml. This concentration range aligns with concentrations documented in patients following conditions such as blunt chest trauma [12]. The mRNA expression and protein secretion of IL-6 and IL-8 increased in a time- and dose-dependent manner (Fig. 2A, B) while the anti-inflammatory adiponectin was significantly diminished (Fig. 2A, B). The morphology of the cells and the size and number of lipid droplets, however, remained unchanged in the adipocyte cultures upon histone treatment (Fig. 2C). To exclude cell strain-specific effects, we repeated the experiment in adipocytes derived from human multipotent adipose-derived stem (hMADS) cells [25] and observed a similar significant upregulation of the inflammatory markers accompanied by a downregulation of adiponectin (Fig. 2D).

### Histone-induced gene activation is mediated *via* a MYD88-IRAK1-ERK signaling axis

**Fig. 3:**
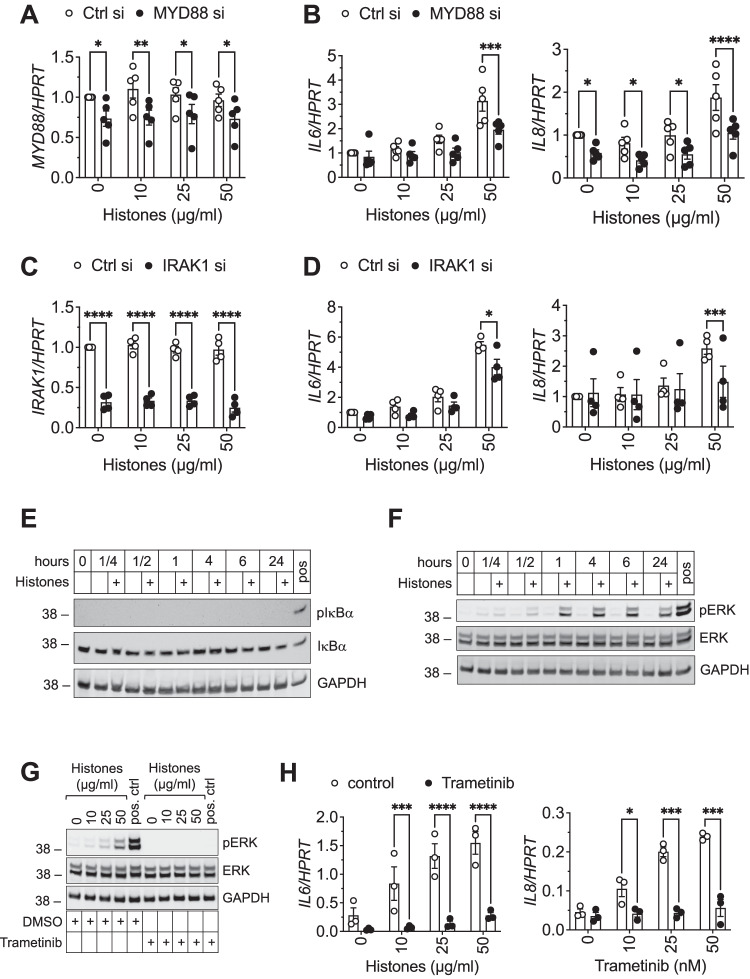
Histone-induced inflammatory cytokine expression is mediated *via* a MYD88-IRAK1-ERK signaling axis. **A**–**D** SGBS adipocytes were transfected with siRNA targeting Myeloid differentiation primary response 88 (MYD88) or control (**A**, **B**) or interleukin-1 receptor-associated kinase 1 (IRAK1) or control (**C**, **D**), and stimulated with increasing concentrations of histones for 4 h. mRNA expression of *MYD88* (**A**), *IRAK1* (**C**), and *IL6* or *IL8* (**B**, **D**) in relation to *HPRT* (2^−ΔCt^) and normalized to Ctrl si without histone stimulation is shown. **E**, **F** Protein was isolated from SGBS adipocytes stimulated with 50 µg/ml histones for the indicated times. Protein expression of phospho-IκBα and IκBα (**E**) and phospho-ERK and ERK (**F**) is shown. GAPDH served as loading control. A549 cells stimulated with 500 ng/ml TNF for 5 min (**E**) or SGBS adipocytes stimulated with 10% macrophage-conditioned media served as positive control (**F**). **G**, **H** SGBS adipocytes were stimulated with increasing concentrations of histones for 4 h in presence of 50 nM trametinib or control (DMSO). **G** Protein expression of phospho-ERK and ERK is shown. GAPDH served as loading control. SGBS adipocytes stimulated with 10% macrophage-conditioned media served as positive control. **H** mRNA expression of *IL6* and *IL8* in relation to *HPRT* (2^−ΔCt^) is shown. Blots are representative of three independent experiments. Data are displayed as mean ± SEM (of five (**A**, **B**), four (**C**, **D**) or three (**H**) independent experiments measured in duplicates). Two-way ANOVA with Bonferroni correction for multiple comparisons, *p < 0.05, **p < 0.01, ***p < 0.001, ****p < 0.0001.

TLRs play an important role in DAMP-induced immune responses in general [37, 38], and in histone-mediated immune responses in particular [39, 40]. In line with our in vivo data, we speculated that extracellular histones may induce inflammatory signaling *via* activation of TLRs in adipocytes. Therefore, we next modulated the expression of two key mediators of the TLR signaling cascade by RNA interference *i.e*., myeloid differentiation primary response 88 (MYD88) and interleukin-1 receptor-associated kinase 1 (IRAK1) in SGBS adipocytes. A knockdown of MYD88 on mRNA level by one third (Fig. 3A) was sufficient to significantly block histone-induced *IL6* and *IL8* mRNA expression (Fig. 3B). Of note, basal expression levels of *IL8* mRNA were also significantly downregulated upon knockdown of *MYD88* (Fig. 3B), in line with previous reports [41]. In addition, knockdown of *IRAK1* (Fig. 3C) significantly prevented upregulation of *IL6* and *IL8* gene expression upon histone treatment (Fig. 3D) in SGBS adipocytes.

Next, we assessed NF-κB and MAPK signaling activation upon histone stimulation which are both further downstream of MYD88 and IRAK1 in the TLR signaling cascade. Surprisingly, histones did neither induce phosphorylation of IκBα nor its degradation (Fig. 3E). However, we found a robust and sustained phosphorylation of ERK1/2 at Thr202/Tyr204 upon histone exposure (Fig. 3F). In line with this, inhibition of the ERK pathway by trametinib, a reversible, highly selective allosteric FDA-approved inhibitor of the kinases MEK1/2, blocked phosphorylation of ERK1/2 (Fig. 3G) and histone-induced upregulation of *IL6* and *IL8* (Fig. 3H). Taken together, our data suggests that extracellular histones induce a pro-inflammatory response *via* a TLR-MYD88-IRAK1-ERK signaling axis in human white adipocytes.

### Free histones induce caspase-independent cell death in human white adipocytes

**Fig. 4:**
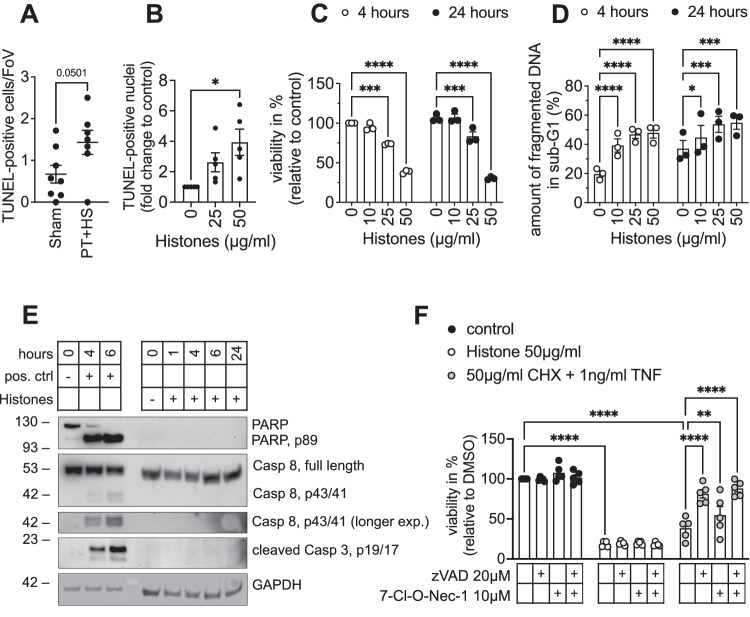
Free histones induce caspase-independent cell death in human white adipocytes. **A** TUNEL-positive cells were counted per field of view (FoV) in iWAT of WT mice with polytrauma (PT + HS) or control (Sham). **B**, **C** SGBS adipocytes were stimulated with increasing concentrations of histones. TUNEL positivity was determined after 16 h (**B**) and viability as assessed by CellTiter-Glo® assay after 4 or 24 h of stimulation (**C**). **D** SGBS adipocytes were stimulated with increasing concentrations of histones. DNA fragmentation was analyzed after 4 and 24 h of stimulation. **E** Protein was isolated from SGBS adipocytes stimulated with 50 µg/ml histones for the indicated times and subjected to analysis by Western blot. GAPDH served as loading control. Nalm6 cells stimulated with 25 µM dinaciclib served as positive control. **F** SGBS adipocytes were pre-incubated with vehicle, zVAD.fmk, 7-Cl-O-Nec1 or a combination thereof for 30 min prior to stimulation with histones or a combination of Cycloheximide (CHX) and TNF as indicated. Data are displayed as mean ± SEM of 7–8 WT mice (**A**), and as mean ± SEM of three (**C**, **D**) or five (**B**, **F**) independent experiments performed in triplicates (**C**, **D**, **F**) or one to three technical replicates (**B**). Unpaired t-test (**A**), one-way ANOVA with Dunnett correction (**B**), two-way ANOVA with Dunnett (**C**, **D**) or Tukey (**F**) correction for multiple comparisons. *p < 0.05, **p < 0.01, ***p < 0.001, ****p < 0.0001. Blots are representative of three independent experiments.

Interestingly, PT + HS resulted in increased TUNEL positivity in iWAT depots in vivo indicative of cell death-associated DNA fragmentation (Fig. 4A). Therefore, we next investigated whether histones also induce cell death in white adipocytes in vitro. Although the cellular morphology remained unaltered (Fig. 2C), we observed an increase in TUNEL-positive nuclei (Fig. 4B) and a loss of cell viability upon stimulation with increasing concentrations of histones in SGBS adipocytes (Fig. 4C). In line with this, the amount of fragmented DNA in sub-G1 [42] concentration-dependently increased (Fig. 4D). Ordered DNA fragmentation is a key feature of apoptosis [43], and extracellular histones are known to induce apoptosis in endothelial cells [44]. Therefore, we assessed molecular key events of apoptosis induction upon histone treatment *i.e*., cleavage of caspases and PARP. We did not observe cleavage of either Caspase-8 or 3 in SGBS adipocytes upon histone stimulation although these two proteins were robustly cleaved in Nalm6 cells treated with dinaciclib serving as positive control (Fig. 4E). Surprisingly, we did not detect any expression of PARP nor its cleavage in SGBS adipocytes, while the positive control revealed both, PARP expression and cleavage (Fig. 4E). This data is supported by the fact that the loss of cell viability could not be prevented by the pan-caspase inhibitor zVAD.fmk (Fig. 4F). Other forms of caspase-dependent cell death such as pyroptosis consequently could be excluded. Necroptosis is a form of programmed cell death which crucially depends on the activity of receptor interacting protein kinase (RIPK) 1. Inhibition of RIPK1 activity by 7-Cl-O-Nec1 did not prevent the loss of cell viability either alone or in combination with zVAD.fmk (Fig. 4F). Of note, both, zVAD.fmk and 7-Cl-O-Nec1 effectively prevented cell death induced by cycloheximide (CHX) and TNF in SGBS adipocytes (Fig. 4F). Taken together, this set of data suggests that cell death observed in SGBS adipocytes upon histone stimulation is neither executed by caspase-dependent cell death such as apoptosis or pyroptosis nor by RIPK1-dependent necroptosis.

### Extracellular histones induce lytic cell death

**Fig. 5:**
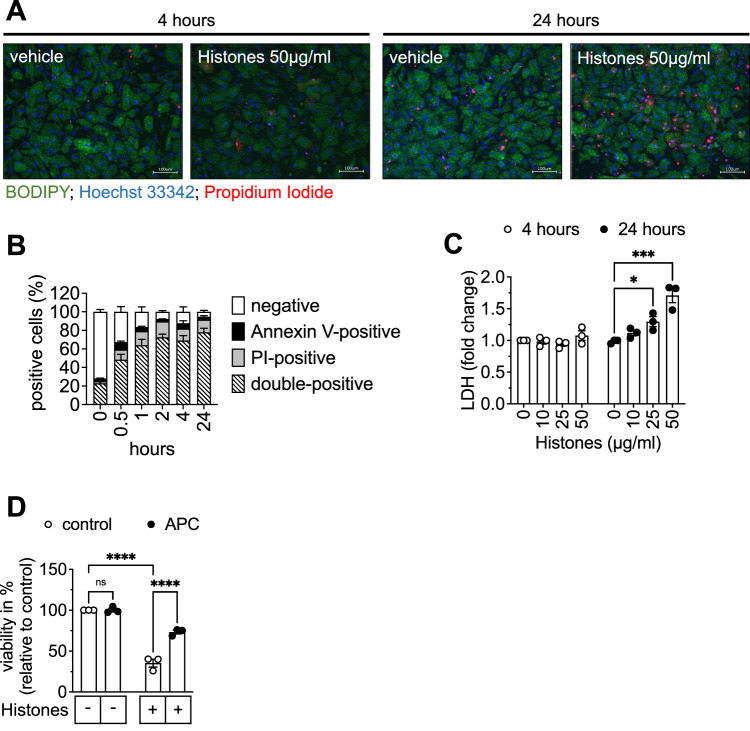
Extracellular histones result in lytic cell death which can be prevented by activated protein C. **A** Representative microphotographs (scale bar 100 µM) of SGBS adipocytes stimulated with vehicle or 50 µg/ml histones for 4 or 24 h and incubated for 10 min with propidium iodide (PI) (red), BODIPY 493/503 (green) and Hoechst 33342 (blue). **B** SGBS adipocytes were stimulated with 50 µg/ml histones for the indicated times, harvested, stained with Annexin-V and propidium iodide (PI), and analyzed by FACS. **C** LDH release into media supernatant after 4 and 24 h is shown. **D** 50 µg/ml histones were pre-incubated or not with 100 nM activated protein C (APC) for 10 min at room temperature before being added to SGBS adipocytes for 4 h. Cell viability was determined by CellTiter-Glo® Assay. Data are displayed as mean ± SEM of three independent experiments performed in singlets (**B**) or triplicates (**C**, **D**). Ordinary one-way ANOVA with Tukey’s multiple comparisons test (**D**). Two-way ANOVA with Dunnett (**C**) correction for multiple comparisons. ns not significant, *p < 0.05, ***p < 0.001, ****p < 0.0001.

Histone proteins are positively charged and can bind to negatively charged cell membranes thereby altering phospholipid membrane permeability [36]. This can lead to lytic cell death through a non-programmed cell death mechanism, as seen in *e.g*. smooth muscle cells [36] or macrophages [45]. To visualize membrane disintegrity, the uptake of propidium iodide (PI) into SGBS adipocytes was analyzed. Here, the number of PI-positive SGBS adipocytes was higher in histone-treated samples compared to controls (Fig. 5A). The PI uptake occurred quickly, and cells did not only stain positive for PI but also for Annexin-V (Fig. 5B), which indicates a rapid loss of membrane integrity upon histone stimulation. Next, we determined the release of lactate dehydrogenase (LDH) into the media supernatant as an additional measure of membrane rupture. Over time, LDH significantly and dose-dependently increased in the supernatant of histone-treated SGBS adipocytes (Fig. 5C).

Activated protein C (APC) is a serum protease known to degrade histones [11]. Thus, we next investigated whether APC could block histone-mediated effects in SGBS adipocytes. Indeed, addition of 100 nM APC significantly reduced histone-induced cell death (Fig. 5D).

### APC and trametinib in combination rescue histone-induced cell death and inflammation in adipocytes and might also be advantageous in a clinical setting of trauma

**Fig. 6:**
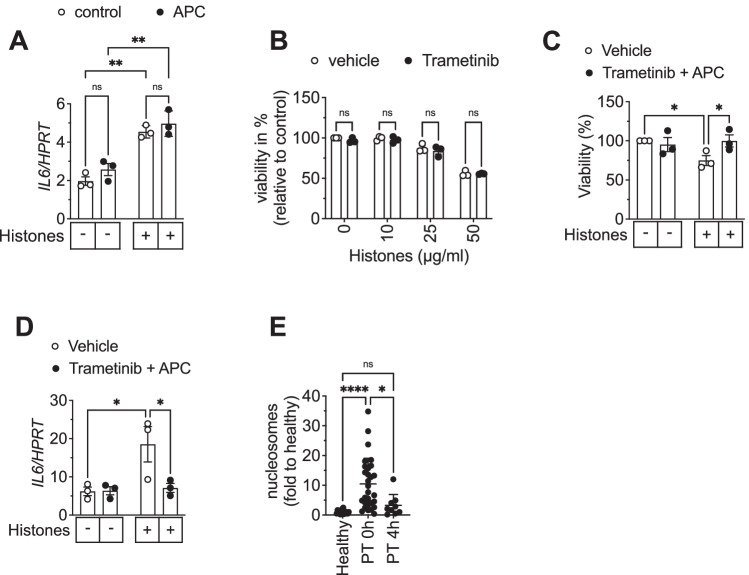
A combination of APC and trametinib rescues histone-induced cell death and inflammation in human white adipocytes and might also be advantageous in a clinical setting of trauma. **A** mRNA expression of *IL6* after 4 h of histone stimulation with 50 µg/ml in presence of vehicle or 100 nM APC in relation to *HPRT* (2^−ΔCt^) is shown. **B** SGBS adipocytes were incubated with vehicle (DMSO) or 50 nM trametinib prior to histone stimulation for 4 h. Cell viability was determined by CellTiter-Glo® assay. **C**, **D** Adipocytes were stimulated with 50 µg/ml histones in presence of vehicle or 50 nM trametinib and 100 nM activated protein C (APC) for 4 h. Cell viability was determined by CellTiter-Glo® (**C**) or mRNA expression of *IL6* determined and depicted in relation to *HPRT* (2^−ΔCt^) (**D**). **E** Increase in fold change of free serum nucleosomes (histones) from human patients after polytrauma (PT) directly at admission to ICU (0 h) and 4 h later compared to healthy control. Data are displayed as mean ± SEM of three independent experiments performed in duplicates (**A**, **D**) or triplicates (**B**, **C**) or as mean ± SD (*n* = 16 (healthy), n = 29 (PT 0 h) and *n* = 9 (PT 4 h)) (**E**). One-way ANOVA with Tukey’s multiple comparisons test (**A**, **C**, **D**, **E**), two-way ANOVA with Bonferroni correction for multiple comparisons (**B**). ns non-significant, *p < 0.05, **p < 0.01, ***p < 0.001, ****p < 0.0001.

To determine whether APC also prevents histone-induced gene activation we assessed expression of *IL6* mRNA upon histone stimulation in presence and absence of APC (Fig. 6A). APC did not block upregulation of *IL6* mRNA upon histone exposure suggesting that also fragmented histones exhibit gene activatory capacity. As trametinib was able to prevent gene activation induced by histones (Fig. 3G, H), we next analyzed whether histone-induced cytotoxicity could be prevented by trametinib. This was, however, not the case (Fig. 6B). Thus, we reasoned that the combination of APC and trametinib might be able to prevent both, histone-mediated cytotoxicity and gene activation. Indeed, the combination of both compounds completely blunted histone-induced cell death (Fig. 6C) and inflammation (Fig. 6D).

To assess the clinical significance of histone release, we determined the concentration of free histones in serum of patients suffering from severe trauma (Fig. 6E). The concentration of circulating histones was elevated by ~10-fold in polytrauma patients at the time of admission to the intensive care unit (defined as 0 h) as compared to healthy subjects (Fig. 6E). Already after 4 h, the concentrations significantly dropped again (Fig. 6E) underlining the fact that a potential treatment with APC and trametinib should be initiated as early as possible after the traumatic incident.

Taken together, this data demonstrates that the combination of trametinib and APC prevents the damaging effects of histone release on WAT and might therefore be beneficial in patients with severe injury, who are at risk of developing trauma-induced metabolic dysfunction.

## Discussion

In this study, we find that histones are released and detectable in increased amounts in the blood in an animal model of PT + HS and in patients with polytrauma, in line with previous publications [8, 12, 46]. Histones are well-known for their role in tissue injury and inflammation [3, 7]. They are released from dying cells and, when detected extracellularly, generate immunostimulatory and even toxic effects in the surrounding tissue [3, 7]. In parallel to the increase in circulating histones we observed an increase in iWAT inflammation after PT + HS. The gWAT depot showed a similar trend, the results, however, did not reach statistical significance.

Adipocytes and their precursor cells, but also various types of immune cells such as macrophages, dendritic cells, B and T cells reside within WAT [16]. All of these cells can be a source of inflammatory cytokines and chemokines such as TNF, MCP-1, and IL-6 [47]. That adipocytes are a crucial source of inflammatory mediators is highlighted by the fact that adipocyte depletion in mice resulted in a substantial reduction in circulating IL-6 during inflammation [48]. We observed that the trauma-induced inflammatory response in iWAT was at least partially dependent on the presence of the TLR co-receptor CD14 as its absence abolished trauma-induced upregulation of *Tnf* and *Mcp1* in vivo (Fig. 1C). Whether the inflammatory response in vivo originates predominantly from tissue-resident monocytes/macrophages or from the adipocytes and their precursors remains unclear at present. However, we demonstrated that stimulation with histones, an important DAMP released after trauma, elicits a strong inflammatory response in two different human white adipocyte strains in vitro (Fig. 2).

Mechanistically, this inflammatory response was dependent on a MYD88-IRAK1-ERK signaling axis (Fig. 3). Histones can induce inflammation also in other cell types [46, 49], and were identified to mediate their effects *via* activation of TLRs [12, 39, 40, 46, 49]. We demonstrated that histone-induced upregulation of inflammatory cytokines and chemokines in human white adipocytes is dependent on MYD88 and IRAK1 (Fig. 3A–D), two central signaling adapters in TLR signaling pathways. Histone-mediated inflammation in adipocytes was blocked by use of the FDA-approved MEK-inhibitor trametinib (Fig. 3G, H). Trametinib was identified to abrogate neuroinflammation after traumatic brain injury in mice [50] suggesting that it can ameliorate trauma-induced inflammation in different organs. Interestingly, however, trametinib failed to specifically block histone-induced adipocyte death (Fig. 6B) whereas activated protein C (APC) was very efficient at doing so (Fig. 5D), in line with previous reports [36, 40, 51]. In septic patients a negative correlation was found between plasma histones and endogenous APC, capable of degrading histones [46]. Recombinant APC (Xigris) was the first biologic agent approved for the treatment of severe sepsis and septic shock. The PROWESS trial reported a reduced 28-day mortality [52]. However, these findings could not be replicated in later trials, leading to the withdrawal of Xigris in 2011. In the aftermath, nevertheless, observational studies have consistently shown a Xigris-mediated mortality benefit [53]. However, based on our data, it is tempting to speculate that APC might be useful in patients with trauma to prevent WAT damage, especially in combination with trametinib to also inhibit the histone-mediated inflammatory signaling axis. Future studies might stratify patients for therapeutic APC application based on histone monitoring. Importantly, a therapeutic intervention should be started as early as possible as circulating histones peaked early in patients after polytrauma.

Trauma patients show increased serum levels of certain adipokines such as leptin, IL-17A and IL-33 [54], triglycerides and free fatty acids (FFAs) [55], the latter two being surrogate markers of adipocyte death [56]. Moreover, triglycerides were elevated in the serum of mice after brain injury [57]. This data suggests that WAT damage and adipocyte death may be a more common phenomenon in trauma patients than previously appreciated. Interestingly, the death of adipocytes was independent of caspase activation and RIPK1 activity (Fig. 4E, F). Although adipocytes did not show any overt morphological alterations upon histone treatment (Fig. 2C), we found them to rapidly become PI- and Annexin-V-positive (Fig. 5A, B) after histone stimulation suggesting lytic cell death to occur.

Patients with polytrauma frequently suffer from metabolic changes such as hyperglycemia and insulin resistance. In the context of obesity, insulin resistance develops on grounds of WAT inflammation [58]. We propose that metabolic sequelae of trauma such as insulin resistance, hyperglycemia, glucose intolerance, and high plasma insulin levels could be due to inflammatory processes in WAT. Therefore, preventing adipose tissue inflammation and adipocyte death could help minimizing metabolic dysfunction in patients with polytrauma.

Patients with severe burn injury undergo browning of WAT and present with elevated energy expenditure and a hypermetabolic state [59]. Patsouris et al. proposed that browning after burn injury is mediated *via* catecholamines [59], which are also responsible for WAT browning upon cold exposure [60]. As adipocyte death was recently suggested to induce the new formation of brown adipose tissue [61] it is possible that also other types of trauma leading to adipocyte death result in WAT browning. In our study, we did, however, not observe any changes in *Ucp1* expression of iWAT in vivo as early as 4 h after trauma (Fig. 1B), yet inflammatory genes were already upregulated. At later time points after a focal surgical trauma of WAT, UCP1 expression was induced at both, the trauma site and the contralateral uninjured site [29]. The effect was first visible after 24 h and persisted for at least 5 days [29]. This illustrates a certain temporal sequence of events. At first, DAMP recognition induces inflammation and cell death in adipocytes. Later, adipose tissue remodeling and browning may occur as described by others [29]. Whether there is a direct link between cell death and browning needs to be clarified in future studies. Nevertheless, inhibition of adipocyte death might prevent WAT browning thereby reducing the hypermetabolic state observed in patients with burn injury [59].

Taken together, our study identified extracellular histones as trauma-induced DAMPs leading to inflammation and cell death in WAT. Histones elicit an inflammatory response *via* activation of a MYD88-IRAK1-ERK signaling axis. In parallel, histone-induced cell death occurs in adipocytes as a non-apoptotic, non-necroptotic form of lytic cell death, which can be inhibited by APC. Inhibition of ERK by trametinib in combination with APC blocked both cell death and inflammation, and might be useful to minimize trauma-induced alterations in white adipose tissue.

### Supplementary information

Supplemental Information

Uncropped Western Blots

## Data Availability

All data generated or analyzed during this study are included in this published article and its supplementary information files.
