# Supplementary material for: Trauma-associated extracellular histones mediate inflammation via a MYD88-IRAK1-ERK signaling axis and induce lytic cell death in human adipocytes
Source: Cell Death Dis. 2024 Apr 23;15(4):285. doi: 10.1038/s41419-024-06676-9 (PMC11039744; doi:10.1038/s41419-024-06676-9)
Supplement: Supplementary file 1 — Supplemental Information [file 41419_2024_6676_MOESM1_ESM.pdf]

Supplemental Information

Trauma-associated extracellular histones mediate inflammation *via* a MYD88-IRAK1-ERK signaling axis and induce lytic cell death in human adipocytes

Roos J [1*], Zinngrebe J [1*], Huber-Lang M [2], Lupu L [2], Schmidt M [1], Strobel H [1], Westhoff MA [1], Stifel U [1], Gebhard F [3], Wabitsch M [4], Mollnes TE [5,6], Debatin KM [1], Halbgebauer R [2], Fischer-Posovszky P [1]

[1] Department of Pediatrics and Adolescent Medicine, University Medical Center, Ulm, Germany

[2] Institute of Clinical and Experimental Trauma Immunology, University Medical Center, Ulm, Germany

[3] Department of Orthopedic Trauma, Hand, and Reconstructive Surgery, University Medical Center, Ulm, Germany

[4] Division of Pediatric Endocrinology and Diabetes, Department of Pediatrics and Adolescent Medicine, University Medical Center, Ulm, Germany

[5] Department of Immunology, Oslo University Hospital and University of Oslo, Oslo, Norway

[6] Research Laboratory, Nordland Hospital Trust, Bodo, Norway

* contributed equally to this work

Running title: trauma response in adipocytes

Corresponding author:

Pamela Fischer-Posovszky

Email: pamela.fischer@uniklinik-ulm.de

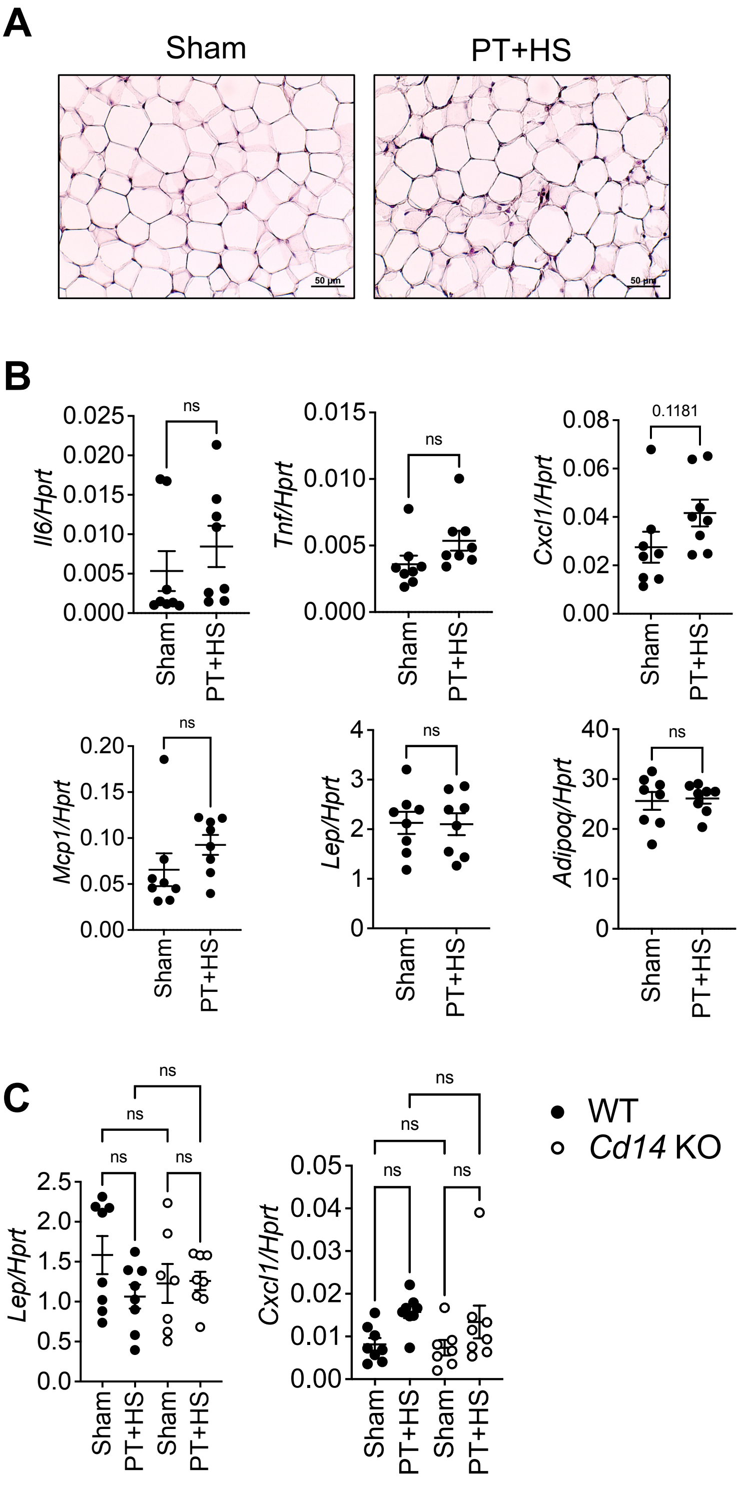

Supplementary Figure 1| Inflammation in adipose tissue after polytrauma with hemorrhagic shock. A – C. Gonadal WAT (gWAT) was collected from mice 4 hours after polytrauma and hemorrhagic shock (PT+HS) or from sham-treated mice. A. Representative

H&E-stained tissue sections from gWAT are depicted. B. *Il6*, *Tnf*, *Cxcl1*, *Mcp1*, *Lep* and *Adipoq* mRNA expression from gWAT is shown in relation to *Hprt* ($2^{-\Delta Ct}$). C. Expression of *Lep* and *Cxcl1* mRNA was determined in inguinal WAT (iWAT) of *Cd14*-deficient mice after PT+HS or sham, and compared to iWAT of WT mice (from Figure 1B). Data are displayed as single values and mean ± SEM of n=8 WT mice per group (B) or n=7-8 *Cd14* KO mice (C) per group. Unpaired two-sided t-test (B), ordinary one-way ANOVA with Tukey's multiple comparison's test (C), ns = non-significant.
