# Supplementary material for: Trauma-associated extracellular histones mediate inflammation via a MYD88-IRAK1-ERK signaling axis and induce lytic cell death in human adipocytes
Source: Cell Death Dis. 2024 Apr 23;15(4):285. doi: 10.1038/s41419-024-06676-9 (PMC11039744; doi:10.1038/s41419-024-06676-9)
Supplement: Supplementary file 2 — Uncropped Western Blots [file 41419_2024_6676_MOESM2_ESM.pdf]

## Appendix – uncropped Western blots

**Fig. 3E** (Note: The membrane from this experiment was first incubated in Tubulin and afterwards in GAPDH which is why remains of Tubulin are found on the GAPDH image.)

pIκBα:

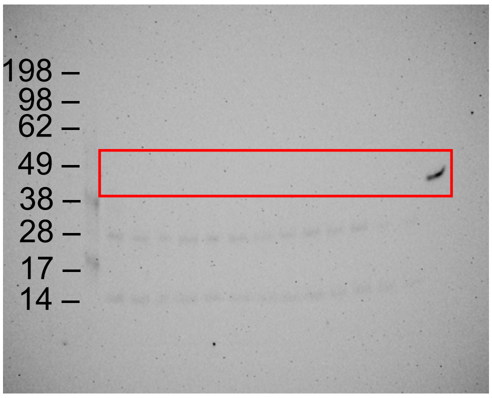


IκBα:

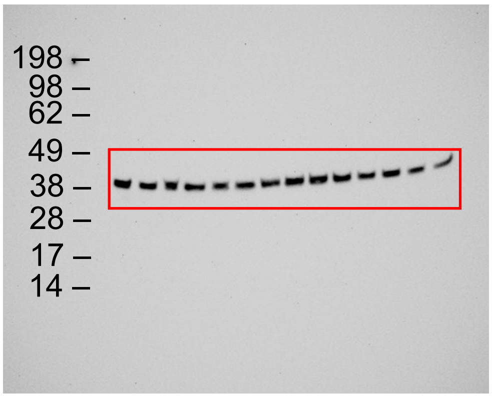


Tubulin (as additional loading ctrl):

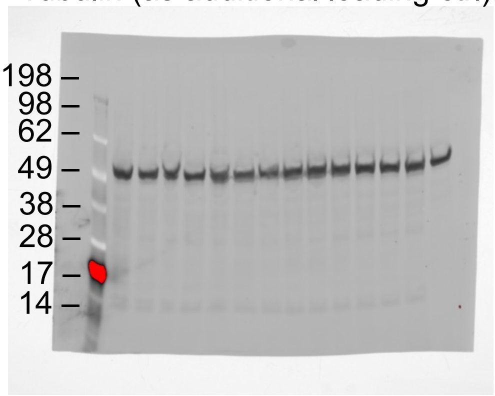


GAPDH (developed after Tubulin):

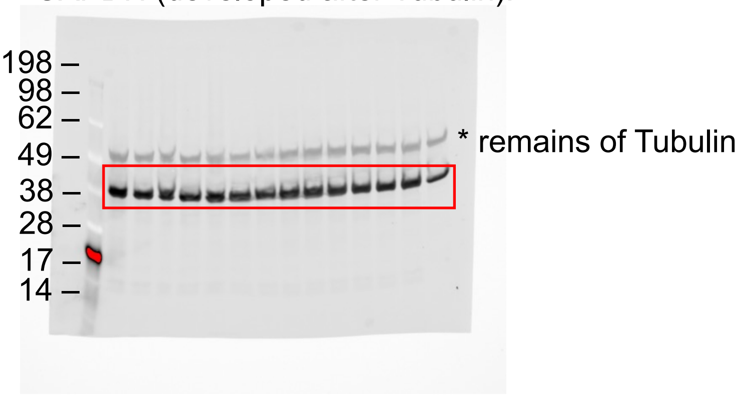

**Fig. 3F** (Note: The corresponding membrane of this experiment was cut (indicated by red arrow) to probe it with different antibodies at the same time.)

pERK:

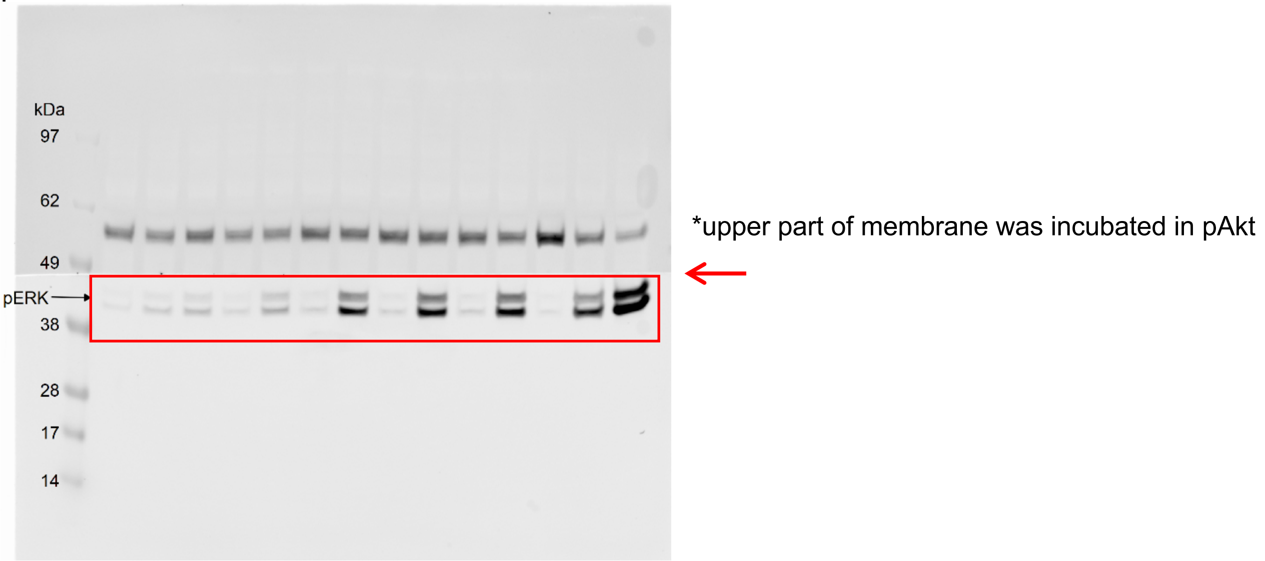


ERK:

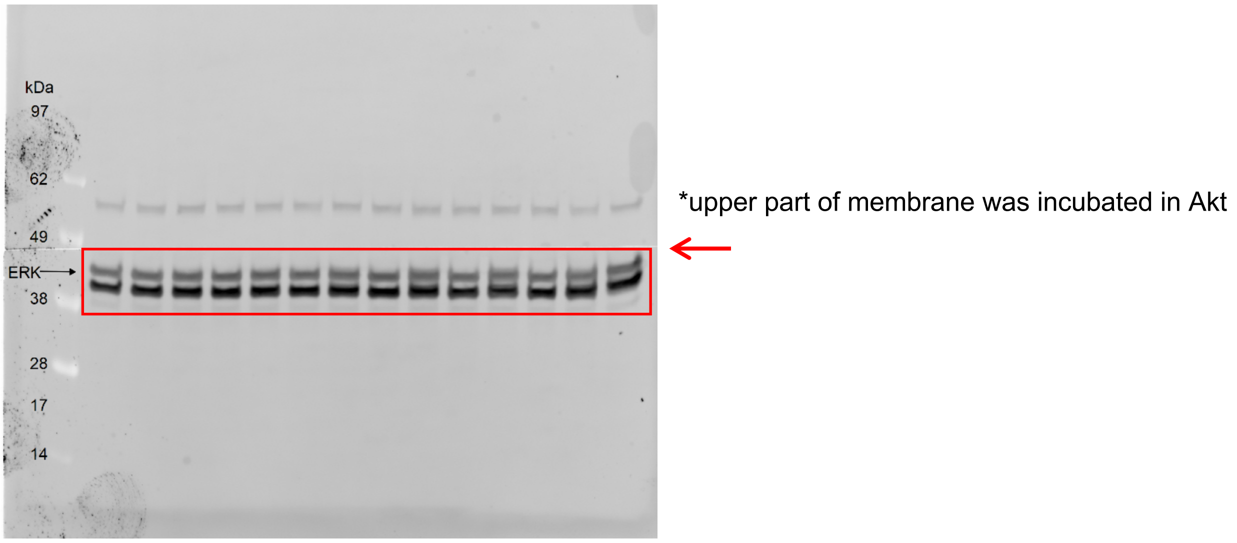


GAPDH:

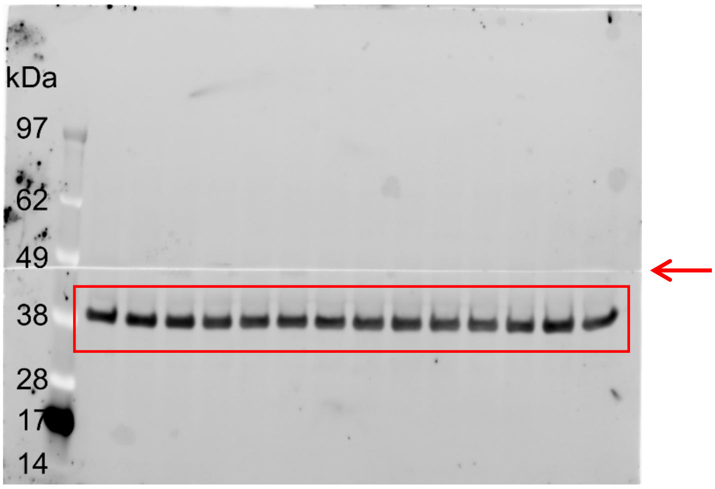

**Fig. 3G** (Note: The corresponding membrane of this experiment was cut (indicated by red arrow) to probe it with different antibodies at the same time.)

pERK:

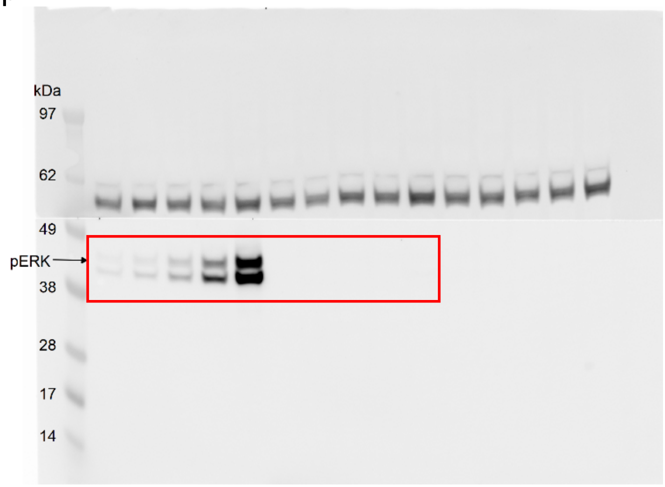


*upper part of membrane was incubated in pAkt

ERK:

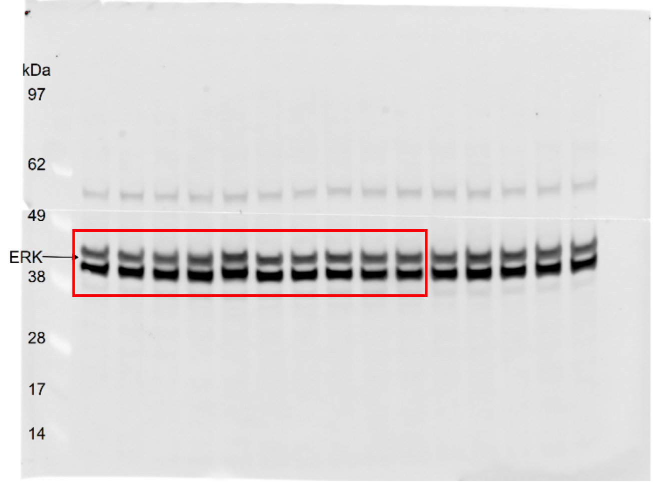


*upper part of membrane was incubated in Akt

GAPDH:

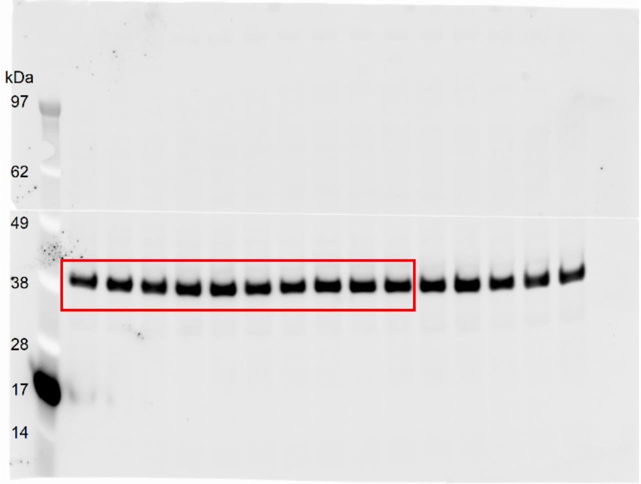

**Fig. 4E** (Note: The membrane from this experiment was partially covered during the imaging process to visualize cleavage fragment of lower abundance. The respective images are marked with an asterisk.)

PARP:

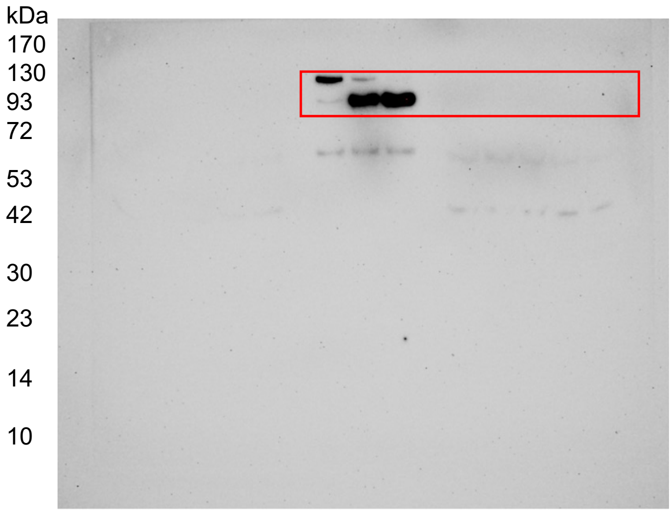


Caspase-8:

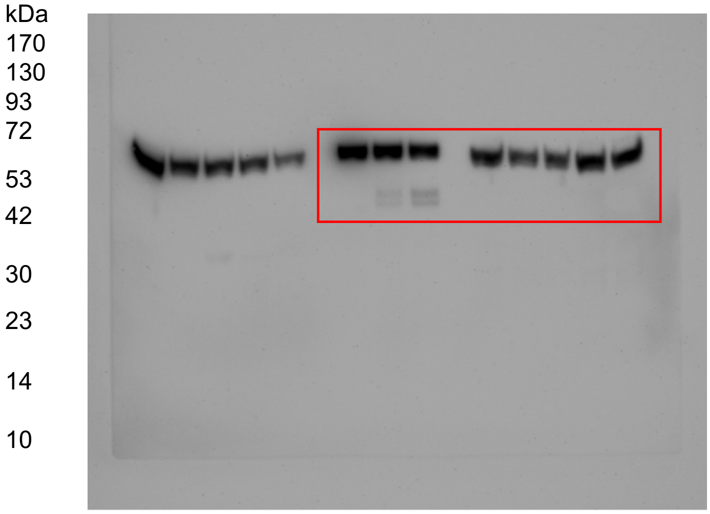


Caspase-8, longer exposure*:

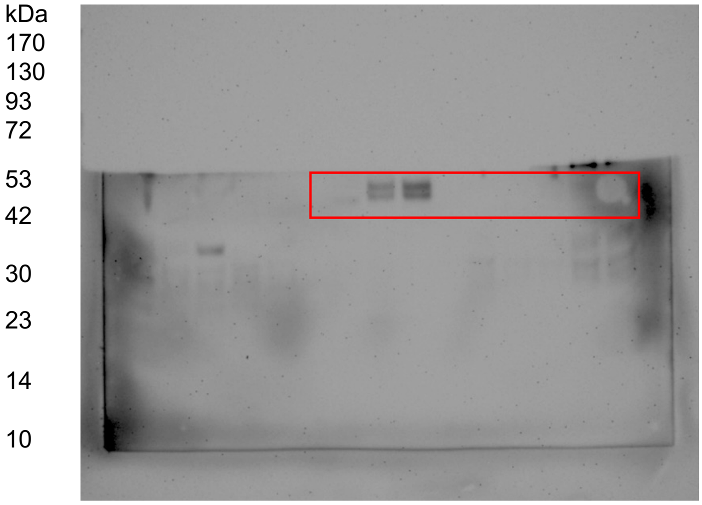

Cleaved Caspase-3*:

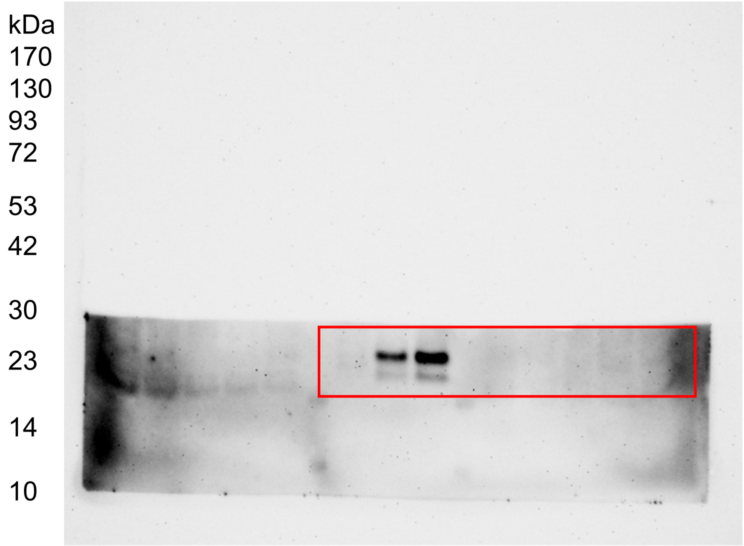


GAPDH:

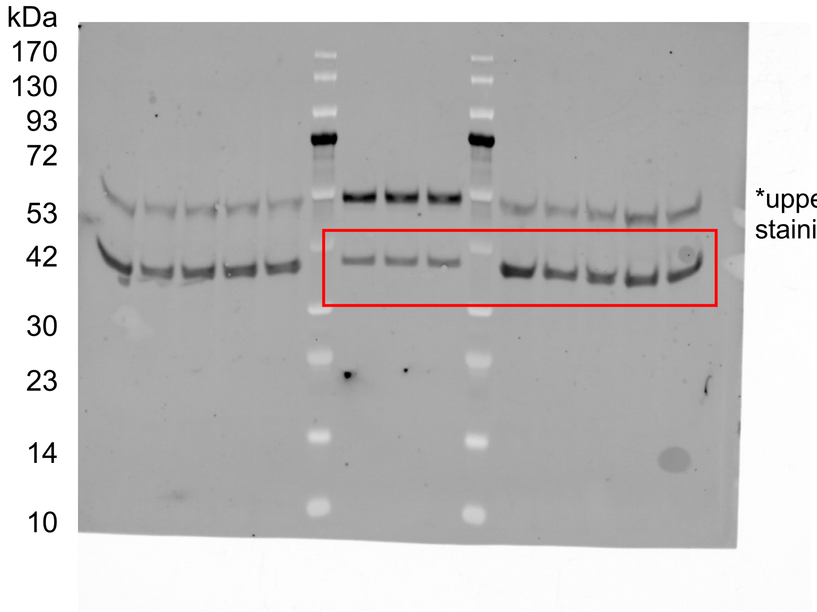


*upper band is from previous staining with anti-Tubulin
